# Grain fields in sea-landscapes

**DOI:** 10.1007/s13280-025-02191-z

**Published:** 2025-05-14

**Authors:** Marieke M. van Katwijk

**Affiliations:** Department of Environmental Science, Radboud Institute for Biological and Environmental Sciences, Radboud University, Heyendaalseweg 135, 6525AJ Nijmegen, The Netherlands

**Keywords:** Blue economy, Climate change, Novel food, Sea level rise, Sea-rice, *Zostera marina*

## Abstract

Sea-level rise will increase salt intrusion and flood risk in low-lying lands. In the long run, these lands will change into seawater-influenced landscapes, as is already happening in several coastal areas around the globe. While conventional agriculture may no longer bear fruit in such sea-landscapes, the seagrass species *Zostera marina*, “sea-rice,” potentially yields 3–7% of global rice production, with the added benefit of zero-carbon emissions. Culture of *Z. marina* does not require freshwater, fertilizer or pesticides. Development and implementation of seagrass mariculture will open new avenues for collaborative efforts of multiple disciplines such as agronomy, coastal engineering and social sciences. From the start, the domestication, engineering design and landscape planning should aim at the optimal balance between ecosystem goods (grains, straw and seafood) and services (coastal protection, carbon and nitrogen sequestration, filtering of pathogens and pollutants, and biodiversity) of this potential crop, while respecting and restoring the wild meadows.

## “Loaves are green and taste well”

Since ancient times, the Seri people of present-day Mexico have known the nutritional quality of the seeds of *Z. marina*, which they used to bake their daily bread (Felger and Moser 1973). In 1974, a workshop was organized in Delaware to inventory potential food crops in a saline environment (Felger and McRoy 1975). It was concluded that culture of *Z. marina* “deserves considerable research effort.” A few years before, colleagues at the University of Arizona had baked a loaf of bread using coarsely ground seagrass flour obtained from Seri people. The group of scientists reported that the seagrass bread was green, had a golden-brown crust and tasted well when fresh, with a flavor that somewhat resembles rye bread (Felger and McRoy 1975). They suggested hybridization with *Z. asiatica* to breed larger seed. To the best of my knowledge, there have been no follow-up experiments, until the recent use of seagrass grains by an haute cuisine chef in Cadiz, Spain (Pérez-Lloréns et al. 2023). The grains served by the chef were cultured by researchers of the University of Cadiz, who additionally determined the nutritional quality of the seeds, finding relatively high protein and mineral content (Pérez-Lloréns et al. 2023). Given today’s challenges of climate change and sea-level rise, the time may have come to further explore large-scale domestication of *Z. marina* for food production.

The area of low-lying lands with enhanced risks of flooding will increase from approximately 540 000 km^2^ of land that is influenced by the sea today (i.e., 1:100 year flooding risk) toward 620 000 km^2^ by the turn of the next century, following a modest warming scenario (1.5 or 2° C warming, Brown et al. 2018). In other words, 80 000 km^2^ may become available for alternative land use (i.e., sea use) toward 2100, even if serious climate action is taken. As a wild species, *Z. marina* already has the biological potential to produce 3–6.5 tons/ha of edible seeds (based on 6.5 mg per seed, 45 000–100 000 seeds/m^2^; van Katwijk et al. 2021), which is similar to domesticated rice species with average yields of 4.7 ton/ha (FAO 2023). Based on the 80 000 km^2^ area, this suggests a production potential of 23–52 Mton, equivalent to about 3–7% of global rice production (FAO 2023). Note that such high seed production in the wild is still exceptional, so breeding to maximize seed production is required to achieve such yields on a large scale.

A key ecological benefit of seagrass meadows is that they attract and support a large faunal community, including invertebrates and juvenile fish. In contrast with terrestrial grain fields, the majority of this fauna will not eat the plants, but rather is attracted by other food sources and the shelter provided by *Z. marina*. Seed predation is common, but at low levels (Carroll et al. 2019). Invertebrates such as shrimps, clams and mussels may in fact also be harvested within seagrass fields, as an additional source of human food. In addition, seagrass fields support adjacent fisheries, as was observed for pollock, herring and cod (Unsworth et al. 2019).

## Zero-carbon emissions and other environmental benefits

Wild seagrass meadows capture and store considerable amounts of carbon, ranging from 50 to 1900 kg C/ha/yr depending on species, environment and climate (McLeod et al. 2011; Marbà et al. 2018). In the first large-scale, successful *Z. marina* seeding project worldwide, a net sequestration of 420 kg CO_2_-eq/ha/yr has been recorded in a 7 km^2^ field, not including emissions from energy use for the seeding efforts and monitoring (Oreska et al. 2020). Energy use and greenhouse gas emissions for seeding, harvesting and local transport for seagrass culture are yet unknown to my knowledge, but to get a feel for the order of magnitude in terrestrial agriculture: European open-field agriculture, still dominantly using energy from fossil fuels, emits about 300 kg CO_2_-eq./ha for seeding, harvesting and local transport (Paris et al. 2022). When also considering decarbonization of the energy and transport sectors into the future, seagrass seed production would likely result in *(sub-)zero-carbon emissions*. Even before planting, there can be carbon emission benefits of the submergence of agricultural land by seawater. For example, a Danish agricultural land emitted 29 000 kgCO_2_-eq/ha/yr prior to submergence, which dropped to zero after inundation by seawater (Petersen et al. 2023).

Seagrass grown as crop would have *no or low fertilizer demand*, as it efficiently filters and recycles nutrients. In fact, excess nutrient addition should rather be avoided than stimulated, to avoid algal overgrowth (McGlathery et al. 2007). Low fertilizer use not only reduces cultivation costs, but also prevents further eutrophication and nitrous oxide emissions. Moreover, existing eutrophication in the surrounding shallow coasts could be reduced by the seagrass nutrient filtering and local storage in the sediments (McGlathery et al. 2007; Moksnes et al. 2021). A related benefit compared to conventional agricultural is that *Z. marina* culture *does not require pesticides* either (Moser and Felger 1973).

**Fig. 1:**
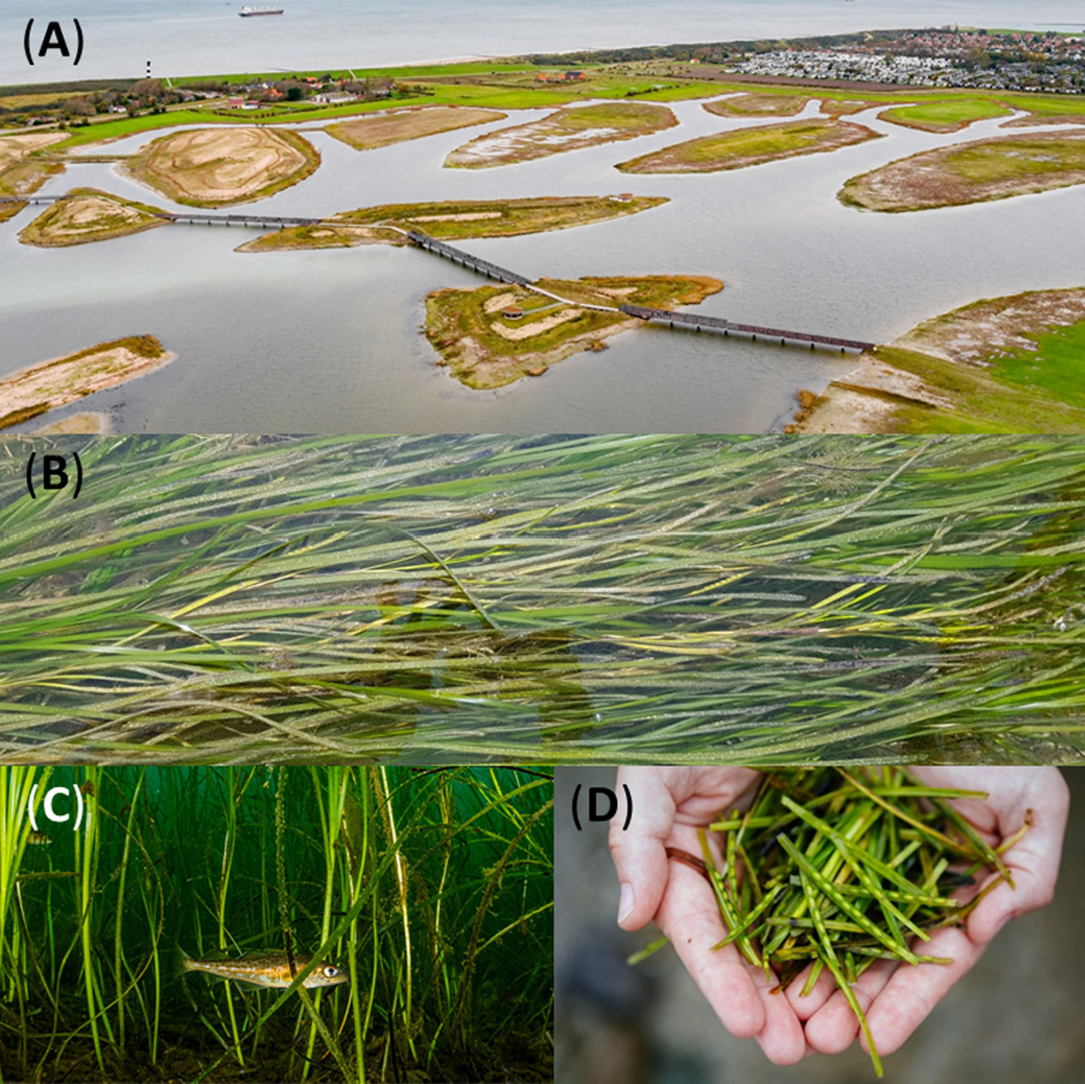
Grain fields in sea-landscapes. Bending the climate change curve? (**A**) Experimental sea-landscape: an inspirational landscape for submarine grain culture (Waterdunen, The Netherlands; Provincie Zeeland, Photo from https://www.zeeland.nl/waterdunen/waterdunen, unknown origin); (**B**) *Zostera marina* leaves and spathes containing seed lying flat during low tide (Gouville, France, Photo by the author); (**C**) baby Atlantic cod (*Gadus morhua*), hiding in eelgrass (*Zostera marina*) Newfoundland, Canada (© Shane Gross, permission granted) and (**D**) flowering shoots consists of 3–20 spathes, each usually containing 6–10 seeds (© Nina Constable/WWF-UK, permission granted)

Seagrass meadows provide a third benefit in the form of *coastal protection*, which they improve via enhanced particle sedimentation that levels up the seafloor. When there is sufficient sediment in the surrounding water, seagrass fields will keep up with sea-level rise and thus help protect the shores. Sedimentation is also a key process in innovative coastal protection schemes currently developed in the Netherlands using double dikes. In a cyclic scheme, segments between the two dikes level are inundated by seawater until they are sufficiently elevated by natural sedimentation, and the segment can be closed off from the sea (van Belzen et al. 2021). Natural sedimentation will be considerably enhanced by the presence of seagrass during the first stages after the seawater enters the segment, and seagrass mariculture could have a rotational scheme in this application. Presently, seagrass is not yet considered in these experimental landscapes, but these Dutch engineering trials could provide excellent living laboratories for further testing and breeding of suitable *Z. marina* strains (Fig. 1).

Lastly, seagrass beds are *natural filters*. They filter nutrients, sediment particles, pathogens, plastics and pollution from the water, thus improving the water quality (reviewed in Unsworth et al. 2022). Part of the filtered material is processed to harmless products in the seagrass microbiome, but another part may be toxic and accumulate. In the latter case, an extra filtering step may be required, for example, by assigning the fringing parts of the seagrass field as a buffer zone.

## An ideal candidate for mariculture

*Z. marina* has several characteristics that could make it an ideal candidate for domestication and mariculture. Harvesting its grains does not require tillage and does not compromise other ecosystem services of the meadow, as the seeds are located well above the sediment surface and out of the way of vegetative shoots, rhizomes and roots. The seed-bearing shoots are in fact positively buoyant, facilitating harvest and subsequent local transport. The seeds themselves, once released, immediately sink to the bottom. Therefore, seed-bearing shoots should be harvested before the seeds are ripe, and brought to tanks for further ripening. The high specific weight of the seeds facilitates further cleaning and separation from lighter debris. The technology for the harvest and cleaning procedure is already developed by the Virginia Institute of Marine Science in the US, to restore a few thousand hectares of seagrass for natural value and the recovery of the gastronomically valuable Bay Scallops (Orth et al. 2020). For further processing to flour, the seeds need to be dried (Felger and Moser 1973).

A second beneficial characteristic of *Z. marina* is that vegetative parts of the plants can also be commercially used (Chubarenko et al. 2021). A large part of the leaves detaches in autumn and wash ashore. They have traditionally been harvested (collected or mown) by cultures across the globe, including in Europe, North America, China and Japan to use as fertilizer, insolation (e.g., in the US Capitol Building, Unsworth et al. 2022), roof cover, cushions and mattresses, and reinforcement of dikes (e.g., in historic dikes in the Netherlands). At present, the processing of beach wreck is not commercial due to legal constraints, plastic pollution and mixture with algae (Chubarenko et al. 2021), stressing the need for improved regulation and filtering.

A third practical advantage is that the plants are facultatively annual, a trait that is only known from rice. This refers to the fact that the plant can employ both an annual and a perennial strategy, and for *Z. marina*, the maximum seed production of perennial populations is nearly as high as those of annual populations (van Katwijk and van Tussenbroek 2023). This plant trait is helpful for breeding and environmental manipulation to tweak the plant to an optimum between seed production (food provision) and vegetative production (leaf material and ecosystem services), making this trait is currently sought after for terrestrial grain production where it does not naturally occur aside from rice (DeHaan et al. 2022; Zhao and Wang 2024).

Fourth, *Z. marina* has a large tolerance to various environmental stressors. It has a high salinity tolerance (salinity 5–40), temperature tolerance (it grows from Greenland to the Gulf of California) and tolerance for high water levels, as long as light is not limited for prolonged periods of time. Wild populations may be adapted to specific salinity, temperature, light levels and other circumstances via phenotypic, epigenetic and/or genetic differences. This variability could initially provide limitations to cultivate individuals from specific populations, but also opportunities for breeding plants with specific traits. The genome of *Z. marina* was published in 2016, and the field of *Z. marina*-omics has been evolving since (Olsen et al. 2016 and papers that cite them).

Fifth, the technology of *Z. marina*-culture does not have to start from scratch; the seed harvesting, processing and seeding have been demonstrated at a relevant spatial scale in Virginia Bays (Orth et al. 2020). Note however that (i) seed production per hectare is relatively low at this location, and (ii) restoration has still low success rates in other parts of the world (van Katwijk et al. 2016). *Z. marina* grain processing for food has been demonstrated at a relevant scale by the Seri people in Mexico, though it is still labor intensive (Felger and Moser 1973) and could require further mechanization and breeding.

## Sea-landscapes

*Z. marina* requires seawater throughflow to avoid algal accumulation and suffocation (Felger and Moser 1973; Felger and McRoy 1975; McGlathery et al. 2007): Some form of landscape engineering should allow for this, either using pumps or the natural tides, perhaps in combination with two seawater entries (two openings in the dike). Waterdunen is a Dutch shoreline retreat project that combines coastal protection with recreation, while facilitating the development of tidal nature and providing an experimental space for mariculture innovation. Submarine grain culture of *Z. marina* could be envisaged in this type of sea-landscapes, which should be upscaled for large-scale production (Figure 1). Abandoned maricultures, as for example are abundant in China, could also provide suitable sea-landscapes for *Z. marina* culture. Globally, a broad spectrum of inspirational sea-landscapes is rapidly emerging—or rather “submerging” (Airoldi et al. 2021). As much as tourists enjoy the sea and sea life, it may require a shift in the human mindset to live and work in a sea-landscape and shift from home gardening to private oyster and mussel culture, as envisaged in new urban futures in Australia (Boström-Einarsson et al. 2022), and catching occasional wildfowl or enjoy hosting a “silver eel hotel” in the back garden. I expect that the Dutch proverb “pump or drown” will be replaced by “pump or adapt.”

The improved water quality and biodiversity provided by the *Z. marina* fields offer clear benefits for other uses of the sea, such as shellfish mariculture and recreation. For example, seagrasses can protect shellfish from pathogens (Dawkins et al. 2024). Conversely, seagrass may benefit from sediment stabilization through off-bottom shellfish culture at the landscape scale. Both seagrass mariculture and shellfish mariculture can be attractive to eco- and gastronomic tourists. The waters will be clear and biodiverse, and special plots may be allocated for the recreational collection of the farmed shellfish and the various crustaceans and other invertebrates in the seagrass (similar to “food forests” on land). Such synergies and diversity across the landscape and between local stakeholders can create new jobs and revitalize local and regional food and landscape systems.

## Planetary health avenues, challenges and opportunities

Creating or using existing sea-landscapes for cereal production can simultaneously provide multiple sustainable food sources and deliver a host of services that benefit local ecosystems, communities and the planet at large. Seagrass mariculture may thus truly improve planetary health. It also creates new avenues for domestication research as well as a testing ground for nature-inclusive engineering. Because it provides so many services simultaneously, and for instance contributes to both climate change mitigation and adaptation, it may also provide a lever for environmental optimism as an antidote to increasing environmental pessimism.

Although large-scale harvesting of seagrass seeds and food processing, as well as the small-scale mariculture of *Z. marina*, have already been demonstrated (Felger and Moser 1973; Orth et al. 2020; Pérez-Lloréns et al. 2023), the path to a new food faces several challenges. First, wild stocks should never be used for food production because large-scale collection of wild seeds would degrade natural gene pools and the resilience and services of wild meadows. Seagrasses continue to decline worldwide and recovery is often unsuccessful due to eutrophication, coastal development, seed unavailability, disturbance activities and knowledge gaps (van Katwijk et al. 2016; Dunic et al. 2021; Unsworth et al. 2023). Habitat improvement is urgently needed. Food production must, therefore, be completely dependent on mariculture, but seed culture technology is still in its infancy and must be developed first (van Katwijk et al. 2021). Second, in line with this, future submersed areas may become unprotected coasts. Unprotected coasts may be challenging for wild seagrass colonization as well as farming. Perhaps even more so than today, as climate change will increase storm frequency and intensity and growing human populations may exert additional environmental pressures on seagrass meadows. Colonization by wild stocks should be preferred here over cultured seagrasses, which may not sustain in such dynamic environments. More generally, restoring wild *Z. marina* stocks worldwide is important to conserve their diversity, ecosystem functions and services, in particular against the backdrop of a changing climate (Unsworth et al. 2022), and should be prioritized over seagrass farming.

Third, high seed production of *Z. marina only* occurs under certain conditions and/or in specific populations (van Katwijk and van Tussenbroek 2023). Therefore, genetic selection and advancing knowledge for the induction of flowering is required.

Fourth, wild stocks may become genetically contaminated by farmed species, requiring measures and regulations. An interesting opportunity arises to also use breeding technology to produce seeds for the restoration of wild meadows. However, care must be taken to avoid genetic selection during seed culture for restoration (Espeland et al. 2017; van Katwijk et al. 2021), whereas, in contrast, seeds grown for food could be the result of selection for traits favorable to farming, such as optimal seed production or nutritional qualities (van Katwijk et al. 2021).

A fifth challenge is that developing breeding technology and breeding strains can take years to decades and large investments. *Z. marina* farming fits well within the concept of the “Blue Economy,” promoting sustainability, social equity and ecosystem services while generating economic activity and resources from the ocean (Bennett et al. 2019; Hasselström and Gröndahl 2021; Rotter et al. 2021). The necessary investments could come from blended finance, i.e., environmental markets and management-based instruments combined with private investments (Hasselström and Gröndahl 2021; zu Ermgassen et al. 2025). However, the blue economy concept and blended finance generally face challenges such as inconsistent public policies, poor coordination (Mazzucato 2013; Christiansen 2021; Cisneros-Montemayor et al. 2021), unjust use of public resources (Hasselström and Gröndahl 2021) and unequal distribution of benefits and harms that can result from unregulated blue growth (Bennett et al. 2019; zu Ermgassen et al. 2025). To address this, diversification, targeted investments and cross-scale cooperation are recommended (Cisneros-Montemayor et al. 2021; zu Ermgassen et al. 2025), as well as risk *and profit* sharing between public and private parties (Mazzucato 2013). Payment for ecosystem services and eco-labeling can also help (Hasselström and Gröndahl 2021). Inclusive and participatory coordination, planning and governance processes are critical to achieving genuine and equitable blue growth (Bennett et al. 2019, 2021; Cisneros-Montemayor et al. 2021).

Sixth, the adoption of *Z. marina* as food source would require compliance with environmental and food safety regulations and potentially adjustment of these regulations (Rotter et al. 2021).

The trajectory toward further development and implementation of seagrass mariculture in the future sea-landscapes will require global collaborative efforts of multiple scientific and technological disciplines such as agronomy, coastal engineering and social sciences, and crucially the support of local communities and local and national decision-makers. Interestingly, as incorporation of seagrass mariculture in policy strategies and landscape designs has yet to begin, the opportunity presents itself to integrate ecological goals and economic activity from the outset (Brandt-Correa et al. 2022). From the start, the domestication, engineering design and landscape planning could aim at the optimal balance between ecosystem goods (grains, straw and seafood) and services (coastal protection, carbon and nitrogen capture and storage, pathogen and pollutant filtering, and biodiversity), while respecting and restoring wild meadows.
